# Moving With Pain: What Principles From Somatic Practices Can Offer to People Living With Chronic Pain

**DOI:** 10.3389/fpsyg.2020.620381

**Published:** 2021-01-25

**Authors:** Emma Meehan, Bernie Carter

**Affiliations:** ^1^Centre for Dance Research, Coventry University, Coventry, United Kingdom; ^2^Faculty of Health, Social Care and Medicine, Edge Hill University, Ormskirk, United Kingdom

**Keywords:** somatic practice, chronic pain, interoception, exteroception, proprioception

## Abstract

This article brings together research from the fields of chronic pain management and somatic practices to develop a novel framework of principles to support people living with persistent pain. These include movement-based approaches to awareness of the internal body (interoception), the external environment (exteroception) and movement in space (proprioception). These significantly work with the lived subjective experiences of people living with pain, to become aware of body signals and self-management of symptoms, explore fear and pleasure of movement, and understand how social environments impact on pain. This analysis has potential to create new ways of supporting, understanding and articulating pain experiences, as well as shaping the future of somatic practices for chronic pain.

## Introduction

Chronic pain is prevalent globally, and it is a major source of suffering (Sá et al., 2019; Treede et al., 2019) affecting an estimated 20% of people worldwide (IASP, 2020b ICD-11). While people with chronic pain describe “being disabled by an experience not visible to others” (Crowe et al., 2019), the challenge for the professionals and practitioners they interact with is in “recognizing the lived experience of a seemingly invisible condition” (Smadar, 2016, p. 108). Although the symptoms may reflect the different sources of pain, there can be common ground among people living with chronic pain. Pain can impact on sleep, capacity to work or travel, family interaction and social activities, meaning people living with chronic pain can become isolated (Jay and Pain UK, 2015). Despite the considerable physical, emotional and social challenges, many people seek to live well each day with their chronic pain.

### Defining Pain

Defining and classifying pain is important as globally recognized and shared understandings can provide the basis for improvements in care, research and health policy decisions. In July 2020, for the first time in 40 years, the most widely accepted and adopted definition of pain was updated to reflect advances in the understanding of pain. This definition represents 2 years of detailed multinational work and consultation by the International Association for the Study of Pain (IASP).

The core IASP definition now states that pain is “an unpleasant sensory and emotional experience associated with, or resembling that associated with, actual or potential tissue damage” (Raja et al., 2020). Core to this new definition is the inclusion of “resembling that associated with” clause, which indicates an appreciation that pain might not always have an obvious biological cause. In addition, this revised definition is accompanied by six key points:

1. “Pain is always a personal experience that is influenced to varying degrees by biological, psychological, and social factors.
2. Pain and nociception are different phenomena. Pain cannot be inferred solely from activity in sensory neurons.
3. Through their life experiences, individuals learn the concept of pain.
4. A person’s report of an experience as pain should be respected.
5. Although pain usually serves an adaptive role, it may have adverse effects on function and social and psychological well-being.
6. Verbal description is only one of several behaviors to express pain; inability to communicate does not negate the possibility that a human or a non-human animal experiences pain” (IASP, 2020a).

The IASP definition of chronic pain states that it is pain that persists or recurs for longer than 3 months. Such pain often becomes the sole or predominant clinical problem in some patients (Bonica, 1953; IASP Taxonomy Working Group, 2011; Treede, 2013). As such it may warrant specific diagnostic evaluation, therapy and rehabilitation. The most recent update of the International Classification of Diseases (ICD-11), approved in May 2019 by the World Health Assembly (IASP, 2020b ICD-11), now recognizes chronic pain as a health condition in its own right.

Of interest to this paper, is the fact that the word “somatic” does not appear in the list of pain terms on the IASP terminology webpage (IASP, 2017). Typically, within health, the term “somatic” is usually considered to be symptoms that pertain primarily to the body. Somatic symptom disorder, for example, describes those who have a range of bodily symptoms, sometimes described as “medically unexplained,” which can be associated with distress and mental health conditions (Henningsen, 2018). On the other hand, the somatic practices discussed in this article are a set of body-mind integrated approaches that work through movement, for overall health and well-being.

### Managing Pain: Mainstream Approaches

People with chronic pain are often in an invidious position when it comes to accessing appropriate specialist support and “onward referral for those patients with unresolved pain is often neglected” (CSPMS, 2015, p. 2). People with chronic pain face protracted waiting times (Burke et al., 2018) and many are unable to access specialist services meaning that their pain remains unmanaged or ignored (Breivik et al., 2006) within mainstream healthcare.

Typically pain management programs offered by specialist pain services have multidisciplinary input and are concerned with a biopsychosocial approach to health and well-being, aiming to support people to “manage their pain and everyday activities better” (The British Pain Society, 2013/2018, p. 4). Many programs involve an education component for which there is a reasonable evidence base (Joypaul et al., 2019). Multidisciplinary teams generally include physiotherapists, psychologists, occupational therapists, nurses, and doctors; while programs use group activities to focus on gentle exercise, mindfulness, cognitive behavioral therapy, and other coping skills. However, even within these relatively well-established “non-pharmacological” approaches which are used within mainstream services, the evidence base of effectiveness is not always robust. This is primarily due to limitations in study design which is reflected in many reviews reporting the low quality of included studies. Typically, systematic reviews reveal a range of limitations such as poor conceptualization of the core focus of the study (Jackson et al., 2019), lack of consensus in measures used (Banerjee et al., 2018), wide range of intervention content (Elbers et al., 2018), methodological bias (Amatya et al., 2018), small sample sizes (Hilton et al., 2016), risk of bias (Anheyer et al., 2019), overestimation of effect (Niederer and Mueller, 2020), and inconsistency in follow-up duration (Devan et al., 2018).

Some pain management programs are run from hospital and others are community-based. Even before the Covid-19 pandemic, aspects of some programs were delivered remotely (Fraser et al., 2019) and there was an increasing turn to app-based options (e.g., Najm et al., 2019). The impact of the Covid-19 lockdown resulted in an immediate need for more reliance on digital technologies and this is likely to be sustained in the future, even though the efficacy or sustainability of this new way of working is unknown (Cameron, 2020).

Other support can be gained through chronic pain advocates, such as Pete Moore’s Pain Toolkit in the UK and Keith Meldrum’s “A Path Forward” in Canada. Arguably pain “management” has negative connotations, suggesting an approach to life improvement, a whipping into shape and organizing an unruly personal experience. On the other hand, Moore promotes self-management, as a means of making decisions and becoming an expert in one’s own needs and capacities; an opportunity to be “in the driving seat” (Moore, 2018 quoted in Goldingay 2018, p. 62). More recently, “supported self-management” has been discussed to indicate that people living with pain are not isolated or alone in the process, but working interdependently with healthcare professionals (Moore and Meldrum, 2020).

### Supporting Pain: Somatic Approaches

Somatic practices encompass a series of movement forms that can be drawn together through their shared focus on body awareness through reflection on movement habits, opening up movement capacity and developing self-directed or personal movement styles. With somatics seen as a form of movement education (Eddy, 2017, p. 7), it highlights “attention to body sensations and interpreting them with a perspective that aims to enhance a quality of life in which one stays present, mindful, even while moving: consciously acting.” Williamson (2010, p. 44) also notes common themes such as self-regulation, pleasurable movement, self-authority, validation of subjective experience, sensory exploration, play and contemplation.

Fortin (2018a, p. 271) argues that there is a “need for further research into using somatic education with people living with a variety of chronic pain conditions and illnesses.” Although there is some evidence that different somatic forms can support people living with chronic pain conditions, addressed later, this evidence is neither joined up nor is it generally well-integrated into public health programs. However, somatic approaches echo some of the activities in chronic pain management programs, such as gaining an understanding of one’s own body through self-awareness and opening up capacity to move in space. Somatic practices can also offer some additional strands such as bringing physical movement in dialogue with mindful awareness in a holistic activity. Somatic practices are also founded in attention to the lived, subjective experience of each person and can explore creative modes of articulating experiences of pain which are difficult to describe. Core to this is the notion of “somatic authority” (Green, 1999) which is underpinned by the desire to take ownership of one’s own life experiences; this is somewhat akin to ideas of self-management. Adopting an approach of somatic authority also echoes (Gilmore’s, 2012, p. 95) argument to see people with chronic pain “as active producers of meaning rather than bundles of recalcitrant symptoms or medical mysteries.”

To date, a set of somatic principles for supporting chronic pain management across a range of conditions has not been theorized or tested, while open structured approaches have been underexplored, and interdisciplinary research on the topic is lacking.^1^ There is some research on structured forms such the Alexander Technique (e.g., Essex et al., 2017; Woodman et al., 2018) and Feldenkrais Method (Ahmadi et al., 2020), while lay books on Hanna somatics for pain exist (Peterson, 2012; St. Pierre, 2015). Open somatic frameworks that “rely on more autonomy in movement response lying with the client” (Weber, 2009, p. 239) with improvisatory, non-stylized guided sessions (such as Authentic Movement) are missing from research on chronic pain. In fact, most other prominent somatic forms are absent from chronic pain research, such as Body Mind Centering; as are semi-structured approaches that are combined with improvised exploration. Tai chi and yoga as practices have distinct bodies of knowledge in relation to pain, and the confines of this article means that we will not address these; our deliberate focus is on contemporarily developed somatic forms.^2^ In particular, we are curious about how a set of principles from somatic practices can be used as cues for open-ended exploration of pain experiences, bringing in the writings of dance and movement artists who work with somatic practices into this article. However, we emphasize that our thinking in this paper aims to open up discussion about its potential contribution to pain management; this is the start rather than the end of the journey. Future research within somatic practices and pain will need to take account of the limitations to study design that have been previously mentioned in relation to other approaches to pain management.

## Background to Somatic Practices in Chronic Pain Management

Dance and somatic practices offer creative knowledge which can inform the improvisation of new ways of working with pain, in response to participants and the settings where work takes place. Implicit in this is a critical reflection on seeing the body as not just something to be treated or cured; it aims to value subjective experience and recognizes that this cannot easily be measured. Mermikides and Bouchard (2016, p. 3) reflect on the history of medicine, with “standardized techniques of assessment, diagnosis and treatment.” They explain that the potential of performance practices to offer “personal reflection and experiential subjectivity” (p. 4) is “contrary to the passive patient who receives care.” Performance practices encourage people to “enact, act out, up or against the passivity” (p. 11–12). However, one of the central tenets of most pain management programs is encouraging people living with pain to play an active part in their own well-being. The underpinning values in somatic practice and multidisciplinary, person-centered programs may be more alike than different, albeit that they may be expressed using different language. Subjectivity exists in both approaches, although somatic practices have specific ways of working with this and articulating it. Dragon (2015, p. 30) notes that: “Somatic education …… supports individuals to pay attention to their internal sensations, to become sensorily self-aware and to use sensed information for the purposes of empowering themselves to make meaning and decisions and to take action in educational, therapeutic, and life situations.” There is real potential for somatic practitioners to bring a depth of thinking and creative exploration about these elements to healthcare professionals working with people living with chronic pain; and indeed directly collaborating with people living with pain on future research.

### Benefits of Somatic Forms to Health and Well-Being

Different somatic forms are reported to have a wide variety of health and well-being benefits for people living with chronic pain. Physical benefits, for example, include flexibility, balance, muscle tone, reduction of days in pain, increased mobility and reduced perception of pain, especially in musculoskeletal pain (Little et al., 2008; Webb et al., 2013; Wenham et al., 2018; Paolucci et al., 2019; Ahmadi et al., 2020). A randomised controlled trial (RCT) of Alexander Technique lessons, exercises and massage for chronic and recurrent back pain found that the effect of 24 lessons in the Alexander Technique after 1 year was a “42% reduction in Roland disability score and an 86% reduction in days in pain compared with the control group” (Little et al., 2008, p. 4). A study on the Feldenkrais Method for osteoarthritis also proved valuable for balance and gait improvement (Webb et al., 2013). Cacciatore et al. (2005) also reported an improvement in tonic muscular activity and postural coordination and pain due to Alexander Technique lessons for people with low back pain. The Feldenkrais Method was included in a comparative study with “Body Awareness Therapy” (BAT) and physiotherapy for non-specific musculoskeletal disorders that included pain (Malmgren-Olsson et al., 2001). Results suggested that BAT and Feldenkrais could be more effective than conventional treatment and BAT achieved a larger improvement in pain severity. Looking more closely, BAT is an approach developed and delivered in a physiotherapy setting which draws on Alexander Technique, Feldenkrais, tai chi and meditation. This suggests that somatic principles shared across forms could be just as effective as distinct forms, and even more effective in an interdisciplinary treatment approach.

Most studies also acknowledge the biopsychosocial elements of pain, though not all. Cacciatore measured only physical approaches such as one-legged standing, kinematics and forces, as well as levels of pain. McClean et al. (2015) gathered quantitative and qualitative data, which concluded that Alexander Technique lessons had an impact on the service users’ perception of pain as well as their capacity to manage pain; while clinicians commented that clients were happier and well-being had improved (Ibid., 2015, p. 6–7). They note that a “key finding of this study is that, while quantifiable changes in pain severity and interference scores were reported, more striking was how service users said they modified their ways of managing the sensations of pain, because of the AT lessons. As such, some service users reported no difference in levels of pain but did report reduction in pain medication and resource use, and consequentially in costs” (Ibid., p. 9). It appears there is a value in developing mixed method approaches which describe the experiences of participants alongside measurable outcomes. Further, it shows the role of somatic practices such as Alexander Technique in supporting self-management, by promoting “‘listening’ to the body,” “self-knowledge” and becoming “more actively involved” in health and well-being (Ibid., p. 9).

### Limitations to the Evidence Base

Although the previously mentioned studies were not systematically selected or reviewed, it is clear that comparison of results is challenging. The studies vary in terms of size and scope, definitions and terminology, the outcome measures used and the interventions employed. The Feldenkrais Method and Alexander Technique dominate the studies, with little attention to creative exploration or open structured forms. Hiding in plain sight is what could be deemed a fundamental design flaw; a majority of studies are led by health and social science researchers but none discussed co-design with dance or somatic researchers. It appears that somatic practitioners were brought in to deliver the chosen interventions, and occasionally some were named as co-authors in publications. Woodman et al. (2018) provide an alternative approach including the use of teacher’s journals in the research, with the lead (health) researcher qualified as an Alexander Technique teacher.

However, dance-based perspectives on chronic pain management are still limited.^3^ The inclusion of dance and somatic researchers in the research design process (and an account of this), would help in understanding how the approach is matched with appropriate methods of data collection, what can and cannot be measured, and, importantly, what counts as evidence. Additional limitations relate to the reporting of the underpinning somatic principles and processes. While there has been a move in the area of somatics in health research to share knowledge, methods and expertise, Fortin (2018b notes, p. 152) that understanding the “how” is significant. She states there is “a need to value and assess the process (the dance intervention) as much as the product (the results of the research).” While a somatic practice session may not be the same for each person or group, an articulation of the process can aid in identifying practices that can be included within health settings, in this case for chronic pain management.

The limitations to the evidence base within somatic practice and chronic pain are not dissimilar to those limitations we presented earlier in relation to other “non-pharmacological” practices, and acknowledgment of these limitation allows us to position the evidence and ideas we present as part of an informed but cautious dialogue.

## Interoception, Exteroception, and Proprioception

In the next sections, we outline three specific interlinked somatic principles of particular relevance to a consideration of how somatic practices may contribute to chronic pain management: interoception, exteroception, and proprioception. We draw on research in dance, somatic practices and in different health and social care professions.

### Interoception

Interoception is a central component of somatic practice, as well as being a topic of research in the field of chronic pain. Interoception is the “sense of the physiological condition of the entire body” (Di Lernia et al., 2016, p. 329) that is generated through “interoceptors located in the internal organs and soft tissues, receiv[ing] sensory information concerning internal, visceral body processes” (Hartley, 1995, p. 122). In somatic practices, participants engage in bringing awareness to the internal body systems and to the information received from them. In Body Mind Centering, for example, this might mean noticing the sensations within muscles (mobility, tension, release), or organs (weight, fullness and so on), including discomfort or pain.

Di Lernia et al. (2016) also point to a complex matrix of interoceptive awareness such as accuracy, sensibility and metacognitive awareness. Research into interoception and chronic pain has found that the “interoceptive matrix plays an important role in pain perception and, supposedly, in chronic pain conditions too” (Di Lernia et al., 2016, p. 331), suggesting need for further research into interoceptive training for people with chronic pain. This might involve increasing accuracy in noticing body cues or decreasing hyper-alertness to overwhelming pain signals. Craig (2003, p. 500) also comments that interoception “seems to provide the basis for the subjective image of the material self as a feeling (sentient) entity, that is, emotional awareness.” This points to the role interoception plays in health and well-being, linking bodily awareness with self-image and emotional experience—pertinent to the physical, emotional, and social facets of chronic pain. Finally, Farb et al. (2015, p. 2) connects interoception to the capacity for “self-regulation,” “connection to the moment,” and one’s ability to effect change’ as an approach to well-being. Interoception is bound up with awareness of and modulating chronic pain experiences on multiple levels, often providing prompts for action such as discomfort or tiredness as cues for changing activity.

At their most basic level, somatic practices draw attention to the internal movement of breath. Brodie and Lobel (2004, p. 82) articulate “the importance of the breath in promoting relaxation, in addition to controlling the effects of the sympathetic nervous system in times of stress.” Somatic approaches can further this practice by moving attention around the body and its systems (such as bones, organs, muscles), and inviting movement expression in response. Interoceptive awareness might, for example, show up a tense stomach or held breath, associated with both physical and emotional experience of pain. The Committee on Pain, Disability, and Chronic Illness Behavior (1987, p. 3) note that “stress and anxiety increase muscle contraction and would thus be expected to exacerbate any pain problem to which this factor contributes. Conversely, any treatment that induces relaxation will reduce muscle contraction and perhaps lessen pain.” Interoception can be the first step toward noticing the ongoing effects of pain, including associated emotional states or social situations with the possibility of exploring ways to respond to these.

Feldenkrais practitioner (Fortin, 2018b, p. 157) notes that “somatics is useful as it is based on each person’s internal sensation, more than on learning specific dance steps…perceiving differences in bodily states is believed to enact changes.” Here, she suggests in somatic practices, it is awareness itself that is a stimulus for change. However, many somatic practices take this a step further by facilitating alternative ways of moving and responding. This might be through exploring subtle physical shifts to accommodate a release of pain and associated emotions, or engaging parts of the body that feel pleasant to move. Movement habits can be changed through somatic re-education and (Hanna, 1988, p. 13) further notes that human “sensory-motor systems continually respond to daily stresses and traumas with specific muscular reflexes. These reflexes, repeatedly triggered, create habitual muscular contractions.” Hanna’s approach is to examine habitual tensions in the body and explore alternatives. Although this approach could be seen as aiming to “correct” the person, it could also be viewed as a means of opening up new options for movement that might shift pain experience and perception. Somatic practices can explore movements unfamiliar to the person in pain, thus providing different sensory information, positions, rhythms or patterns.

We propose that somatic approaches can work with interoception by attending to body systems, the sensations or feelings arising from them and by exploring these cues through movement. Attention on the pain itself, such as reading cues for when rest or pacing are needed, may be helpful at times. Mehling et al.’s (2013, p. 413) study note that people who are mind-body trained (yoga, mindfulness/meditation, Alexander, Feldenkrais) tend to distract themselves less often from pain or discomfort and argue that “mindful interoceptive awareness can be an advantageous coping style for pain and discomfort.” This training was found to promote greater awareness “of the connection between body sensations and emotional states” and that their participants “listen[ed] more often to the body for insight, and experience[d] their body more often as safe and trustworthy.” Inherent to somatic practices is supporting people to listen to and trust body experience and notice different sensations and movement supports. This does not mean solely focusing on the regions with pain, but sensing the body as a whole. For example, in Becker et al.’s (2018, p. 85) study of people with neck pain, they note that Alexander Technique considers the person as a whole focusing “on building awareness and integration through the whole musculoskeletal system.” Body Mind Centering practitioner Dowler (2013) also describes how in somatic approaches the body is not compartmentalized into body parts that are painful, tested and repaired but the focus is on valuing the sensed, subjective experience of the individual as a whole person. Dowler’s thinking is very different to pharmaceutical approaches that center on medication fixing the pain and which sustain and potentially amplify a compartmentalized approach.

However, the focus on sensory stimuli can be overwhelming for some people. Valenzuela-Moguillansky et al.’s (2017, p. 1) study on interoception in people with fibromyalgia showed a “higher tendency to note bodily sensations,” but a “lower tendency to actively listen to the body for insight.” This means that painful sensations become heightened, while trusting body cues for self-regulation is reduced (2017, p. 10–11). Borg et al. (2015, p. 42) also note “heightened interoceptive awareness” in people with fibromyalgia, which they associate with hypervigilance. This increased attention or vigilance toward stimuli is likely tied up with the sensory and emotional effects of continued pain (2015, p. 40, 42). Attention to interoceptive experience alone may not enough, especially as this might heighten focus on painful experience and (Farb et al., 2015, p. 5) suggest that a central question to be considered is “understanding how to skilfully relate to interoceptive sensations, and under what circumstances they should be attended to.” Somatic practices for chronic pain therefore should attend to the individual needs of each person, focusing on or reducing attention to pain when helpful, as a form of modulating interoception. This could take the form of transferring attention to pleasurable movement, or interactive/social movement, or engaging in playful, motor activity as a form of shifting sensations and feelings.

### Exteroception and Proprioception

Somatic practices also focus on exteroceptive awareness of the environment, to prompt action or movement in space. Olsen (2002, p. 57) notes that the: “exteroceptors, found in the skin and connective tissue, are responsible for monitoring the outer environment through “touch,” including several kinds of sensations such as pressure, heat, cold, pain, and vibration.” Further, Olsen describes how “proprioceptors, found in the joints, ligaments and tendons, muscles, and the inner ear, are cumulatively responsible for registering movement, balance, and body position in space.” Proprioception is therefore considered as an awareness, perception or sensation of movement and position. The interconnectedness between interoception, exteroception, and proprioception is apparent. Cues from the external environment could impact on the internal body systems such as muscles, and then result in movement in space. Interestingly, the overlap between interoception and exteroception in registering pain is described by Craig (2003, p. 304). The sensation of pain is not only an interoceptive function (sensed in internal body systems) but can be stimulated through touch from the environment. These processes of interoception, exteroception, and proprioception are not conceived as completely separate in somatic practices, rather, shifting attention can provide more detailed information, especially in less dominant modes of awareness for that participant. However, here we group exteroception and proprioception together since they serve joint functions in somatic principles for chronic pain—in engaging with the surrounding environment through movement in space.

Tsay et al. (2015, p. 221) note that people with chronic pain display “altered sensitivity to exteroceptive stimuli” for example being unable to discern the location of touch on their body or changed perception of the size of their body. It is surmised that this arises from pain intensity taking up attention, which also causes hypervigilance to pain and increased sensitivity for example to sounds, smells or tastes. They also suggest that people with chronic pain can have less awareness of proprioceptive signals from the body, in terms of effort or balance for example. Likewise (Valenzuela-Moguillansky et al., 2017, p. 6) note that people with fibromyalgia “described feeling a larger body over the course of a pain crisis” which led to them underestimating their capacity to move through tight spaces. This points to altered exteroceptive or proprioceptive cues, which can prompt a “guarding-type” mechanism toward the body (Ibid., p. 8). Sensory, somatic and embodied approaches are identified as possible supports for people living with pain to work with these challenges (Tsay et al., 2015, p. 221; Valenzuela-Moguillansky et al., 2017, p. 12). As yet, the processes that these embodied approaches could take for chronic pain specifically is not clear.

Chronic pain can increase fear of movement and guarding against further pain; people take efforts to “avoid further aggravation” (Tufnell, 2017, p. 20). Lamé et al. (2005, p. 15) discuss what is described as “fear-avoidance” understanding of pain, noting that “These models assume that pain catastrophizing promotes fear of movement/(re)injury. The latter, in turn, leads to avoidance behavior, disuse, disability and depression.” If a person is living with chronic pain, the worry of worsening it can be sufficient to stifle movement. Pain management programs encourage gentle movement activity to counteract this concern. Tufnell (2017 notes, p. 20) that “slowing down, connecting gently to the rhythm of breath and visualizing slow movement can be a powerful tool in restoring a sense of ease and capacity to self-manage.” We add to this that bringing curiosity to exteroception and proprioception are a means by which fear of movement might be explored.

Brodie and Lobel (2004, p. 82) identify how somatic practices work with exteroception and proprioception through a series of activities in “sensing the environment” and “connectivity” between body and space. Becoming aware of the environment is seen provide the “proprioceptive system with information necessary for accurate and appropriate reaction to stimuli.” A somatic approach to pain management could integrate cues from the both the internal body systems and environment to instigate movement in space. Reeve (2018, p. 76) notes how her approach developed from Amerta Movement, a somatic form cultivated by Javanese artist Suprapto Suryodarmo, explores “paying attention literally to how I shape myself within the structural aspects of my chosen environment: how I position myself in standing, sitting, walking, lying down or crawling and how I become somatically aware of how that position affects and is affected by the world around me.” This movement exploration which engages exteroception and proprioception could support someone living with pain to identify how they relate to their surrounding environment, noticing where fear causes avoidance and testing out possibilities for movement in space.

Somatic practices therefore might prove beneficial to encourage people back into appropriate levels of movement, facilitating through attending to the comfort levels of participants. Visualization of movement is important during pain flare-ups where it can be difficult to feel motivated to move, even gentle movement explorations that can be done in any body position. The studies on somatic practices we identified did not indicate any contraindications against specific somatic forms in working with people living with pain. For example, a review of “Evidence-Based Non-pharmacologic Strategies for Comprehensive Pain Care” notes that Alexander Technique and Feldenkrais Method are safe, with low adverse effects (Tick et al., 2018, p. 194), while Little et al. (2008) also found that “no significant harms were reported” in using Alexander Technique for chronic back pain. At the same time, we encourage working with exteroception and proprioception so that over-stimulation is not reached. Especially, this would be the case when pain sensitivity levels are high during flare ups, where adhering to the notion of modulating rather than awareness overload would be helpful.

Working through a somatic approach brings the opportunity to extend from internal body awareness to connecting with space, in an understanding of habits and fears associated with movement. An aim would be to increase confidence of moving in space, extending the range of movement available where appropriate. A somatic exploration could also aid in understand how environment or context impacts on people with pain. A Bristol-based group of artists who have experience of persistent pain, The Unchartered Collective (2020) and founder member, dance artist Raquel Meseguer, are addressing how public spaces need to be adapted. They have explored the potential to change cultural institutions, such as running a “horizontal cinema” which offers alternative viewing options for those who cannot sit for long periods without pain or discomfort. Meseguer (2018) has also spoken about how public spaces could be designed better for people living with pain, in her advocacy for public resting spaces.

Somatic practices focus in detail on exteroception and can add ways of bringing attention to and describing the environmental factors in pain experience. In future research, we will examine how somatic principles support this process of translating personal pain experiences into a form of public agency.

## Discussion

Although some health-lead chronic pain management does adopt relational approaches (Chow and Fok, 2019; Lew and Xin, 2020), somatic practices with their concerns about subjective experience, self-regulation (Williamson, 2010), self-management or authority (Green, 1999) can offer a new perspective to chronic pain research. This is because chronic pain research has traditionally been led by health researchers whose lens is perhaps more narrowly focused on psychological and/or pharmacological interventions (Birnie et al., 2020) or focused on concepts such as pain catastrophizing (de Oliveira-Souza et al., 2019) which is different from the thinking and values underpinning somatic practices. While some research exists on somatic practices for different forms of chronic pain (such as fibromyalgia or musculoskeletal pain), none address in detail the somatic principles to support chronic pain management, nor do they examine in detail the open-structured and creative elements. Within mainstream health research evidence gaps exist particularly in relation to patient-oriented priorities (Birnie et al., 2020). We propose that somatic practices may be one way in which such gaps may be addressed and a more patient-centric perspective adopted. However, well-designed, robust studies are needed to create an informed evidence-base. These studies will need to take account of the criticisms and limitations that are evident in existing literature addressing non-pharmacological interventions for chronic pain management to ensure design factors such as appropriate sample sizes, clear conceptualization, carefully chosen outcome measures and appropriate duration of follow-up are taken into account. At the same time, what is considered evidence needs to be addressed in working with dance and somatic practices, especially when gathering data on subjective experiences that are not easily measured.

In this article, we have discussed what somatic principles of interoception, exteroception, and proprioception can offer those living with chronic pain and consider how somatic practices are underpinned by being holistic, creative and facilitated approaches. We bring concepts from chronic pain and somatic practices together to create a novel framework for engaging with people with chronic pain. This could build on existing work within health that is developing evidence-based, mind-body programs to increase physical functioning for people living with chronic pain (Greenberg et al., 2019), bringing in body-mind perspectives (Eddy, 2017, p. 12).

Through interoception, or internal body awareness, a person living with pain could learn coping strategies such as becoming aware of body cues for rest or pacing. Interoception could help to relax the nervous system, and therefore tensions or emotions that increase pain. Modulating attention is important—sometimes attuning to pain experience, and other times bringing attention to pleasure in the body, but always seeing the person as a whole and not a set of painful body parts. Interoception promotes valuing subjective body experience (Dowler, 2013) and to developing trust in insight from the body as a way to self-regulate (Mehling et al., 2013).

Fear of movement can restrict people living with chronic pain (Lamé et al., 2005; Tufnell, 2017; Fowler et al., 2019; Simons et al., 2020). The somatic processes of attention to the environment in exteroception, and registering movement, balance and position in proprioception allow the person living with pain to explore movement in space, especially with regard to fears of movement. Somatic approaches can support sensing the environment, exploring triggers for fear, and developing strategies for moving with confidence. These principles can therefore support awareness for both personal and social purposes, in terms of recognizing disabling environments.

However, much research needs to be undertaken and we offer recommendations for further research. Interdisciplinary research on somatic practices should examine the subjective experiences of chronic pain with particular attention to more open-ended improvisatory somatic approaches. Such creative experimentation may be well-suited to responding to changing needs in people living with pain.

Self-regulation and modulation through attending to body cues is core to somatic practices. This raises questions about how research can monitor the hypothesized increased awareness of these cues and how they become integrated over time into daily activities. In future, this could include measuring the development of interoceptive awareness, changes to perception of space, or increased movement scope. Fini et al. (2014) note that the effort required to traverse space changes perception of how near or far away it appears. For someone living with pain, the discomfort and fatigue experience will no doubt affect perceptions of and motivations for movement in space. Since perception and action are linked, recording participant perceptions of space alongside whether movement in space has increased through somatic practices would be valuable. Further, whether social interaction between peer participants and/or the somatic practitioner influences perception of and action in space would be important to understand, as (Fini et al., 2014, p. 9) proposes, “when sharing the extrapersonal space with other bodies, a wider portion of space appears as near and therefore accessible^4^.”

However, questions arise around whether process-oriented somatic movement can be monitored, predicted and measured in the same way as goal-oriented movement or exercise programs (e.g., following set movements within a set program)^5^. Somatic practices are not aiming toward a set form in the movement, but rather exploring experiences, so evaluating errors of “performance” might not be the best way to understand the impact of the practices. Instead, what is valuable to record is the capacity to track habits and experiences, to try new movement behaviors, where appropriate, and in that way, to self-regulate. Including arts-based approaches for gathering such data, such as images, gestures, writings or videos as a form of self-reflection, could support understanding of how people with pain are integrating insights from the practices.

An equal collaboration between health, dance and somatic researchers would create the basis for integrative research design that draws on both arts and health approaches to data collection and dissemination, which acknowledges different disciplinary expertise and ways of perceiving the world. Working collaboratively would ensure credence to different knowledge bases that would ensure the findings offer authenticity across different fields and could provide a broader view on what somatic practices can offer.

Further, language around the benefits of somatic practices for people living with pain might convey the idea that there is something “wrong” with people that needs to be improved. Future research will need to ensure the participation of people with lived experience to guide the process of what is needed and barriers to engagement. Patient and Public Involvement and Engagement is an important and growing aspect of health research. While working directly with people’s experiences is central to somatic practices, it is especially important to consider how people with lived experiences of pain are incorporated into research design and not just in monitoring the results that emerge from somatic practices. People living with chronic pain could therefore identify what is supportive, without undermining the authority and agency they have as experts in their own values and needs. And finally, it is also essential to record any limitations of somatic approaches for people with different types of and outcomes from chronic pain. As (Fortin, 2018b, p. 160) notes, attention needs also to be given to negative experiences, as a means of understanding what does not work or what could be developed further.

## Conclusion

The fields of somatic practices and health could greatly potentially benefit from a closer working relationship in the area of pain management. New ways need to be found to support the millions of people worldwide who live with chronic pain (Goldberg and Mcgee, 2011). These methods need to be meaningful to the people living with pain and we propose that they should include an increased focus on subjective, experiential aspects. Exploring somatic principles of interoception, exteroception, and proprioception are ways in which people living in pain could develop both their awareness and modulation of sensation, emotion and environments associated with pain (and pleasure). If these invisible aspects of chronic pain experiences can be well-understood and articulated, there is potential for this to be helpful to a person living with chronic pain—in their family life, as well as in healthcare systems, workplaces and public spaces.

Somatic practices offer new ways of thinking with and about chronic pain. In best practice, somatic practitioners see people living with chronic pain not as patients needing to be rescued or changed but as agents to collaborate with in ways that promote their well-being—which further can deepen and develop the practices themselves.

## Author Contributions

EM and BC contributed to the conception of the article and to researching and writing sections and manuscript revision. Both authors read and approved the submitted version.

## Conflict of Interest

The authors declare that the research was conducted in the absence of any commercial or financial relationships that could be construed as a potential conflict of interest.

## Footnotes

1 See Weber, 2009 for further information on structured vs. open somatic frameworks.

2 We would, however, like to acknowledge the important influence of yoga and tai chi practices and philosophies on contemporary somatic practices, and that many practitioners consider them part of a somatic practices as a field. Also outside of the remit of this article is Dance Movement Psychotherapy and Somatic Experiencing both of which are fields of research and practice which come from different backgrounds and have their own knowledge base.

3 There are a few exceptions such as Erber’s (2015) informal qualitative study on chronic pain using her Dance Movement Psychotherapy and somatic therapy trainings (as well as her lived experience of pain), and Fortin’s (2018a) research on the Feldenkrais Method for women with Fibromyalgia. However, we focus on principles of somatic practices that can be used across somatic forms for supporting people with chronic pain, in an interdisciplinary approach.

4 The other bodies mentioned by Fini et al. (2014) can include virtual avatars, however, the human form must have the potential to move in space to support changing perception of space (Fini et al., 2015). This opens up alternative avenues for research into the role of virtual somatic practices for chronic pain.

5 For more information on monitoring and predicting action, especially in paired or group activity, see Vesper et al. (2010) and Moreau et al. (2020).
